# rAAV9‐mediated supplementation of miR-29b improve angiotensin-II induced renal fibrosis in mice

**DOI:** 10.1186/s10020-021-00349-5

**Published:** 2021-08-18

**Authors:** Ju-hong Zhang, Jing Li, Yang Ye, Wang-qi Yu

**Affiliations:** 1 The Affiliated Hospital of Hangzhou Normal University, No. 1 Wenzhou Road, Gong Shu District, Hangzhou, 310016 Zhejiang People’s Republic of China; 2 Department of Cardiology, Sir Run Run Shaw Hospital, Zhejiang University, Hangzhou, 310016 Zhejiang People’s Republic of China

**Keywords:** Collagen, Extracellular matrix deposition, Recombinant adeno-associated virus, Renal interstitial fibrosis, miR-29b, Gene delivery

## Abstract

**Background:**

Renin–angiotensin–aldosterone system activation is the critical factor in renal remodeling and dysfunction. Our previous study suggested that miR-29b may attenuate AngII-induced renal intestinal fibrosis in vitro. In the present study, we aimed to determine whether recombinant rAAV9-mediated miR-29b delivery protects against AngII-induced renal fibrosis and dysfunction.

**Method:**

Mice were treated with AngII via osmotic mini-pumps, or phosphate-buffered saline. rAAV9 vectors were produced using the rBac-based system in SF9 cells. rAAV9-miR-29b or rAAV9-control-miR was injected into the kidneys of mice subjected to the model of AngII infusion. The role of miR-29b in renal fibrosis was assessed using quantitative polymerase chain reaction, western blot, and histology.

**Results:**

In AngII-induced fibrotic kidney tissue, miR-29b expression was downregulated. rAAV9-miR-29b delivery significantly reversed renal injury as indicated by decreased serum creatinine and injury related gene expression in AngII-infused mice. Regarding organ remodeling, tubulointerstitial fibrosis and deposition of extracellular matrix components such as collagen type I and type III were significantly decreased in renal tissue from mice delivered rAAV9-miR-29b.

**Conclusion:**

Our results demonstrate great potential for use of rAAV9 as an applicable vector for delivery of miR-29b as an antifibrogenic factor for treatment of tubulointerstitial fibrosis-induced renal injury.

**Supplementary Information:**

The online version contains supplementary material available at 10.1186/s10020-021-00349-5.

## Background

Renal interstitial fibrosis is a primary feature of numerous chronic kidney disorders, and it contributes to end-stage renal failure. Multiple extracellular matrix (ECM) components, particularly collagen type I and III, play crucial roles in interstitial fibrosis (Zeisberg and Neilson 2010). Collagen expression often correlates with the severity of interstitial fibrosis. Therefore, inappropriate collagen accumulation is an important therapeutic target in renal fibrosis. Experimental evidence shows that tubular epithelial cells lose their epithelial features gradually before fibrosis, which leads to the epithelial-mesenchymal transition (EMT). Activation of renin-angiotensin II-aldosterone system (RAAS) signaling—the classical risk factor for tissue remodeling—is a key mechanism involved in kidney remodeling (Almeida et al. 2012). Therefore, in addition to essential etiological control, antifibrosis treatment has been considered as an approach to protect renal function. Because of limitations of RAAS blockers (including side effects such as renal impairment and aldosterone escape) for treating chronic kidney disorders, prevention of renal fibrosis is far from satisfactory.

MicroRNAs (miRs) regulate gene expression by promoting mRNA degradation or translation inhibition. Emerging evidence shows that miRs play pathological roles in kidney diseases. Many studies demonstrated that miRs are either affected by RAAS inhibitors or target RAAS effectors (DuPont et al. 2016). Therefore, miRs may represent novel therapeutic targets for RAAS overactivation-induced kidney remodeling.

In our previous study, we found that miR-29b was downregulated in the kidneys of spontaneously hypertensive rats and AngII-stimulated NRK52E cells, suggesting it may protect against fibrosis by suppressing deposition of ECM components and preventing the EMT (Pan et al. 2014). Among miRs, miR-29b regulates the highest number of collagen genes and genes associated with ECM (Liu et al. 2010). Bretschneider et al. (2016) reported that activation of the mineralocorticoid receptor (MR) reduces the levels of miR-29b in vascular smooth muscle cells. MR activation is therefore a risk factor for tissue fibrosis. Additionally, MR antagonists are limited in their capacity to prevent renal fibrosis because of clinical side effects. On the basis of these findings, we hypothesized that miR-29b may serve as a novel therapeutic target for renal interstitial fibrosis by inhibiting ECM deposition. To test this hypothesis, we used a baculovirus-insect cell expression system to deliver synthetic pre-miR-29b into mouse kidneys. Using a mouse model of renal interstitial fibrosis induced by AngII infusion, we observed ameliorating effects of miR-29b overexpression on renal interstitial fibrosis and dysfunction.

## Methods

### Generation of recombinant adeno-associated virus serotype 9 vector

Recombinant adeno-associated virus serotype 9 (rAAV9) vectors were produced using the rBac-based system in SF9 cells as previously described (Chen 2012). rAAV9 vectors were packaged with single-stranded DNA containing the enhanced green fluorescent protein (eGFP) gene under control of the mouse cytomegalovirus (CMV) and U6 promoter (AAV9-CMV-U6-eGFP). Vector titers were determined using quantitative polymerase chain reaction of vector genomes.

### In vivo gene transduction using rAAV9 vectors

All animal experiments were performed in accordance with guidelines of the Institutional Animal Care and Use Committee of Zhejiang University. Wildtype C57/BL6J mice were purchased from the Shanghai Institute of Biological Science. Eight-week-old male mice were divided into four groups (n = 6 per group). After mice were fully anesthetized by injection of 50 mg/g of 0.8% pentobarbital, the left kidney was exposed and injected with rAAV9, 1*10^10^ infectious viral particles/site at five sites. Kidneys were injected with vehicle, rAAV9-random miR, or rAAV9-miR-29b. Additional animals that underwent the same procedure with injection of phosphate-buffered saline served as age-matched controls.

### Identification of in vivo transduction efficiency

Transduction efficiency of rAAV9 vectors in mouse kidneys was assessed according to GFP expression, measured using both western blot and fluorescence analysis. Once kidney tissues were harvested, fresh frozen sections at 5-μm thickness were immediately prepared. GFP expression was assessed using fluorescence microscopy (Leica, Germany). At least three images at 200 × magnification were captured from each section. The Image J 32 software package was used for analysis.

### Statistical analysis

Data are presented as the mean ± standard error (SEM). Normality was tested using Shapiro–Wilk test. Statistical differences among groups were determined by Student t tests for comparisons between 2 groups and one-way ANOVA tests for multiple groups. All analyses were performed using GraphPad Prism 6, *P* < 0.05 was considered statistically significant and we corrected significance of multiple comparisons using Tukey’s multiple comparison test. Images were processed using Image J, Adobe Photoshop CS5, and Adobe Illustrator CC 2017 software. The adjudgment and semi-quantitative analysis of each slice were performed by two independent investigators in a blinded manner.

A detailed description about the materials and methods is available in Additional file 1, which includes the following parts: mouse model, Western blot analysis, histology immunohistochemistry, terminal deoxynucleotidyl transferase dUTP nick end labeling assay, real time polymerase chain reaction.

## Results

### Administration of rAAV9 restores miR-29b gene expression in vivo

**Fig. 1:**
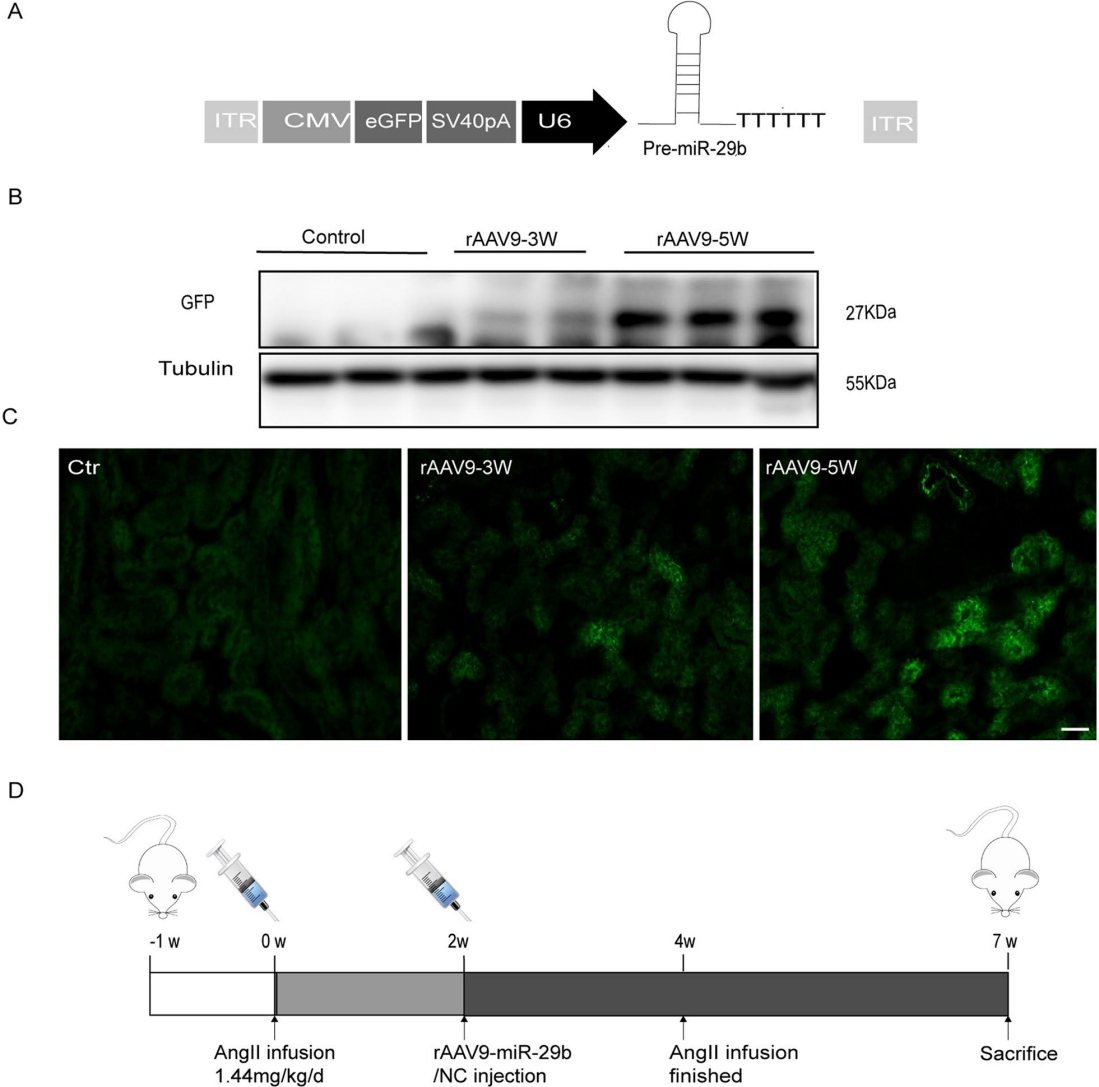
Design of the rAAV9 mediated miR-29b transduction in vivo. **A** Schematic representation of miR-29b expression cassette. The expression cassette was comprised of miR-29b stem-loop sequence flanked by its native intron sequence, which preserves the putative hairpin structure and proper endogenous processing. A genomic fragment 800 bp containing the miR-29b precursor was PCR amplified from the mouse miR-29b precursor sequence and cloned into the self-complementary rAAV9. miR-29b under the control of cytomegalovirus and U6 promoter. rAAV9-GFP transduction in renal tissue was tested by GFP **B** western blot and **C** fluorescence in fresh frozen sections of kidney. In AAV-injected kidneys, there is significant EGFP fluorescence in tubule epithelial cells at 5 weeks. **D** Overall design of the study in vivo. Mouse were subjected to continuous AngII or PBS infusion. Two weeks later, when fibrosis was evident, the animals were randomly chosen to receive either recombinant adeno-associated vector type 9 carrying miR-29b (rAAV9-miR-29b) or control vector (rAAV9-random miR) at 5*10^10^ IVP per kidney via in situ injection

A genomic sequence with pre-miR-29b was PCR amplified from the sequence of the mouse miR-29b precursor and cloned into the sequence-complementary AAV genome vector with the U6 promoter (Fig. 1A). In our preliminary experiments, rAAV9-GFP particles were injected into mouse kidneys, and GFP expression was measured to evaluate the efficiency of rAAV9 gene transduction. Animals were sacrificed 3 or 5 weeks after injection for quantification of GFP expression. GFP expression was robust at 5 weeks, and higher than at 3 weeks. Transduction efficiency was confirmed by western blot analysis (Fig. 1B) and microscopy of stained fresh frozen sections (Fig. 1C).

### miR-29b gene transfer reverses fibrosis and prevents functional deterioration

**Fig. 2:**
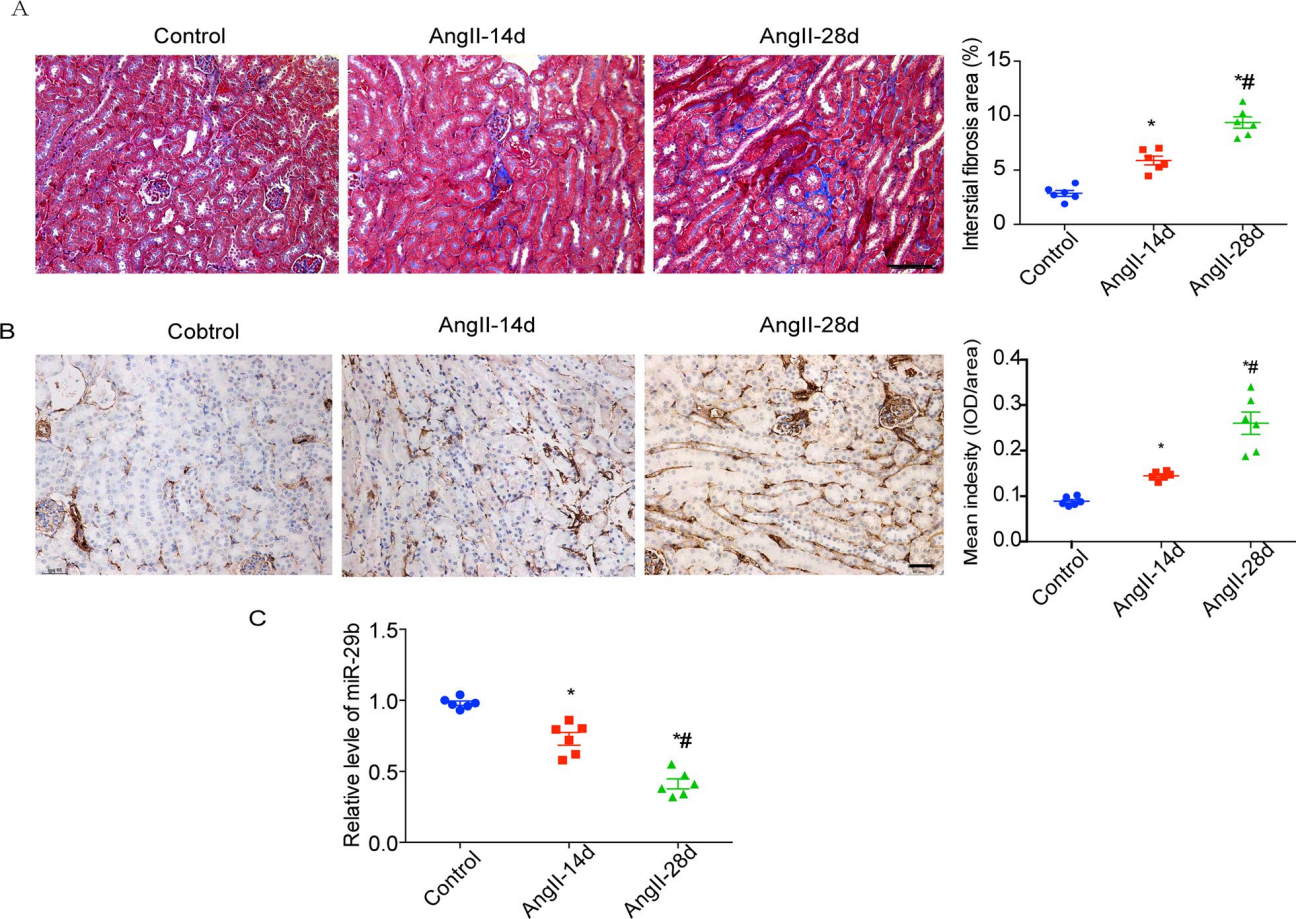
Decrease of miR-29b levels in mouse kidney during angiotensin II-induced renal fibrosis. Representative images of Masson trichrome staining (**A**) and a-SMA immune stain (**B**) of kidney with saline or angiotensin II infusion at different time points. Magnification, × 200. Right histogram represents quantitative analysis of tubular interstitial fibrosis. Results are presented as mean ± SEM. ^**^P < 0.01 compared with control group. ^#^*P* < 0.01 compared with angiotensin II infusion group (Day 14). Each group consisted of six mice. Scale bar 50 μm. **C** Real-time PCR further confirmed the decrease in miR-29b levels in renal tubular epithelium among different groups. Control, mice were treated with saline. AngII-14d, mice were treated with continuous angiotensin II infusion for 14 days. Ang II-28d, mice were treated with continuous angiotensin II infusion for 28 days. ^*^P < 0.05 compared with control group. ^#^*P* < 0.05 compared with angiotensin II infusion group (Day 14)

Increased concentration of AngII was shown to modulate fibrosis via direct effects on the ECM and by regulating expression of other factors (Valls-Lacalle et al. 2018). To clarify the role of miR-29b in renal interstitial fibrosis, we used AngII infusion as a model of renal interstitial fibrosis. In a preliminary experimental model, we observed significant fibrosis following continuous AngII infusion for 28 days (Fig. 2) As shown in Fig. 2A and B, anti-α-smooth muscle actin (α-SMA) and Masson trichrome staining indicated that, compared with the control group, AngII-infused mice all demonstrated features of renal interstitial fibrosis. As shown in Fig. 2C, real-time PCR further confirmed the decrease in miR-29b levels in renal tubular epithelium of the fibrotic kidney (Fig. 2C).

**Fig. 3:**
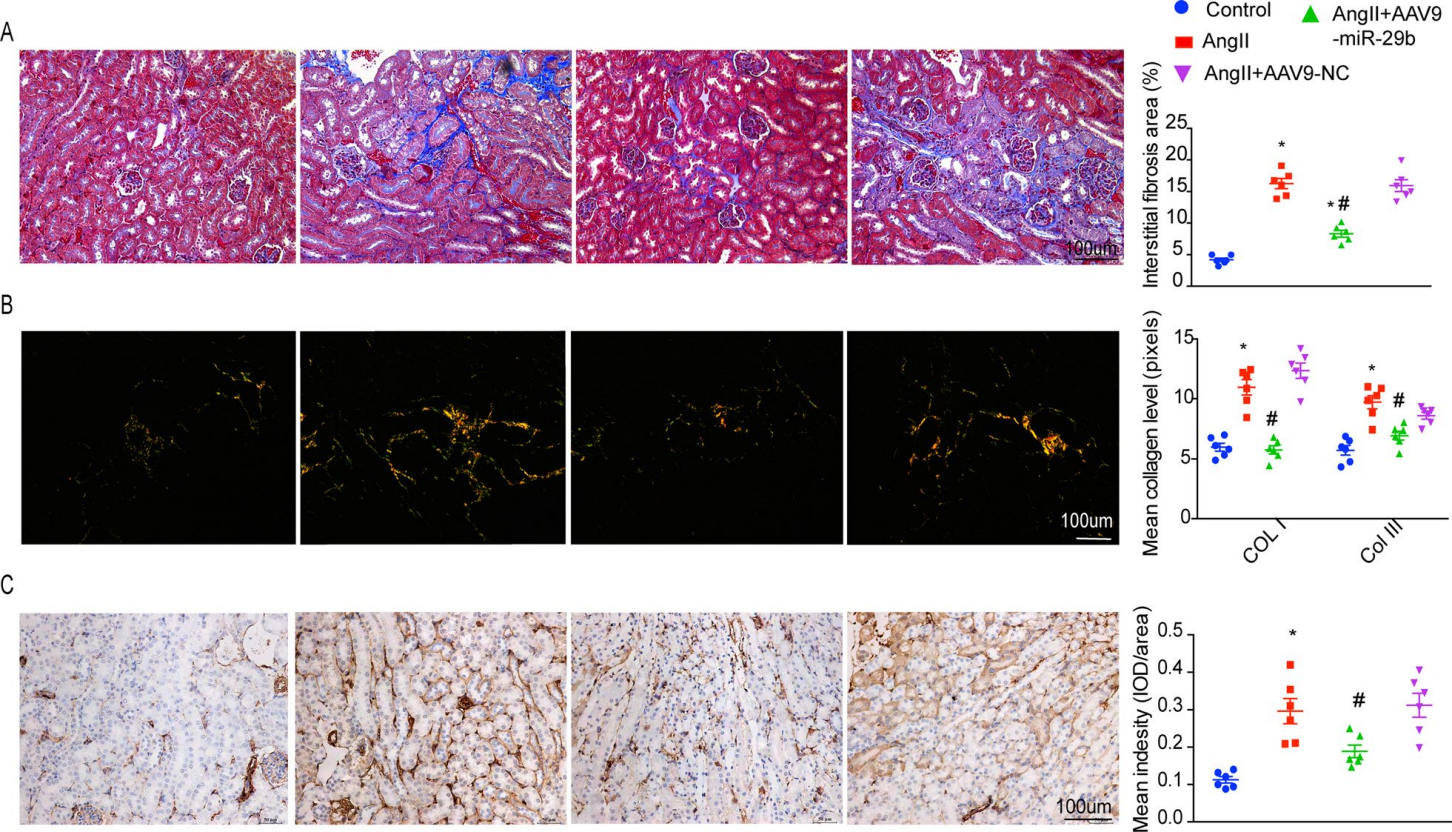
Effects of rAAV9-miR-29b transduction on fibrotic changes in AngII-induced kidneys. **A** Representative Masson staining of kidney sections in each group quantitative analysis of fibrotic areas. Areas with positive blue staining show collagen fibers. Magnification, × 200. **B** Representative sirius red staining of kidney sections and quantitative analysis of fibrotic areas. Area with yellow stain show collagen type I (COL I) and green stain show collagen type III (COL III). Magnification, × 200. **C** Representative images of alpha-smooth muscle actin (a-SMA) immune staining of kidney among the groups(six high-power fields counted per group). Magnification, × 200. Values are mean ± SEM, n = 6. *P < 0.05 compared with control group. ^#^*P* < 0.05 compared with rAAV9-negative control. Scale bar 100 μm

On the basis of these results, we hypothesized that miR-29b protects against AngII-induced renal interstitial fibrosis. To test this hypothesis, we delivered rAAV9-miR-29b into mouse kidneys via in situ injection to determine if this ameliorates AngII-induced downregulation of miR-29b. Delivery of rAAV9-miR-29b effectively increased the levels of miR-29b in mouse kidneys. As shown by Masson trichrome, Sirus red, and anti-α-SMA staining of mouse renal tissue sections in Fig. 3A–C, respectively, delivery of miR-29b-expressing vectors significantly ameliorated tubular interstitial fibrosis and EMT induced by AngII. These results suggest that delivery of miR-29b can significantly prevent renal interstitial fibrosis.

### Effects of miR-29b on fibrosis markers and profibrotic signals in the kidney

**Fig. 4:**
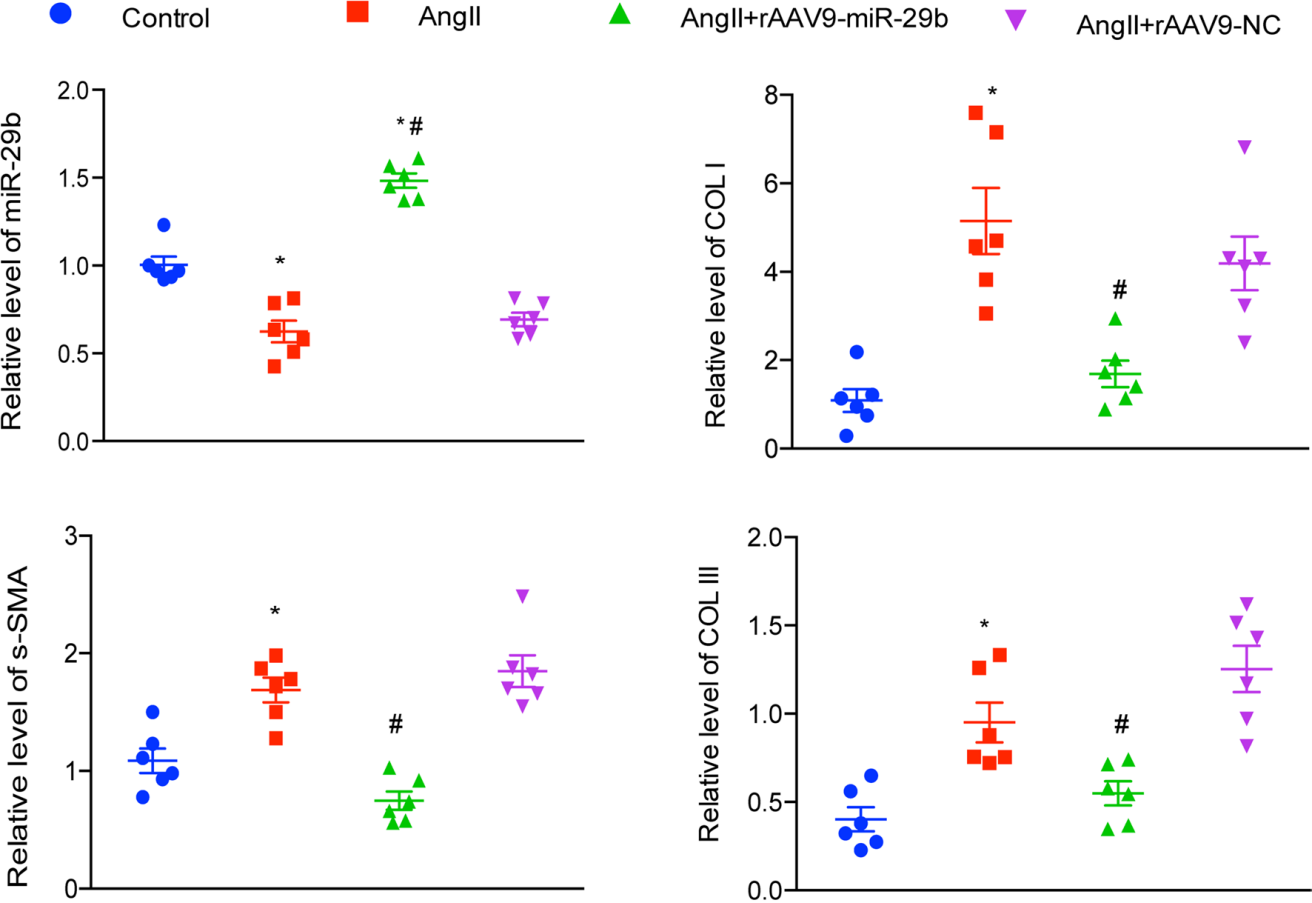
Effect of rAAV9-miR-29b delivery on fibrotic markers in kidney. Mature miR-29b levels was significantly increased in rAAV9-miR-29b transduction group. The expression levels of alpha-smooth muscle actin (a-SMA), collagen type I (COL I) and collagen type III (COL III) were evaluated as fold change relative to the control animals at 5 weeks post gene transfer. Values represent mean ± SEM (n = 6). Significance of differences: *P < 0.05, rAAV9-miR-29b vs. control group, ^*#*^*P* < 0.05, AngII + rAAV9-miR-29b vs. rAAV9-negative control. rAAV9 indicates recombinant adeno-associated vector type 9; NC, negative control

As shown in Fig. 4, delivery of miR-29b-expressing vectors significantly mitigated the reduction of miR-29b in mouse kidneys following AngII infusion. However, delivery with an miR mimics control vector did not have the same effect. qRT-PCR analysis indicated that expression of α-SMA, collagen type I, and collagen type III was increased in AngII-infused kidneys. Expression of these markers was significantly inhibited by rAAV9-miR-29b transfer, but not by negative control, − rAAV9-miR (Fig. 4).

### Effect of rAAV9-miR-29b delivery on renal injury induced by AngII infusion

**Fig. 5:**
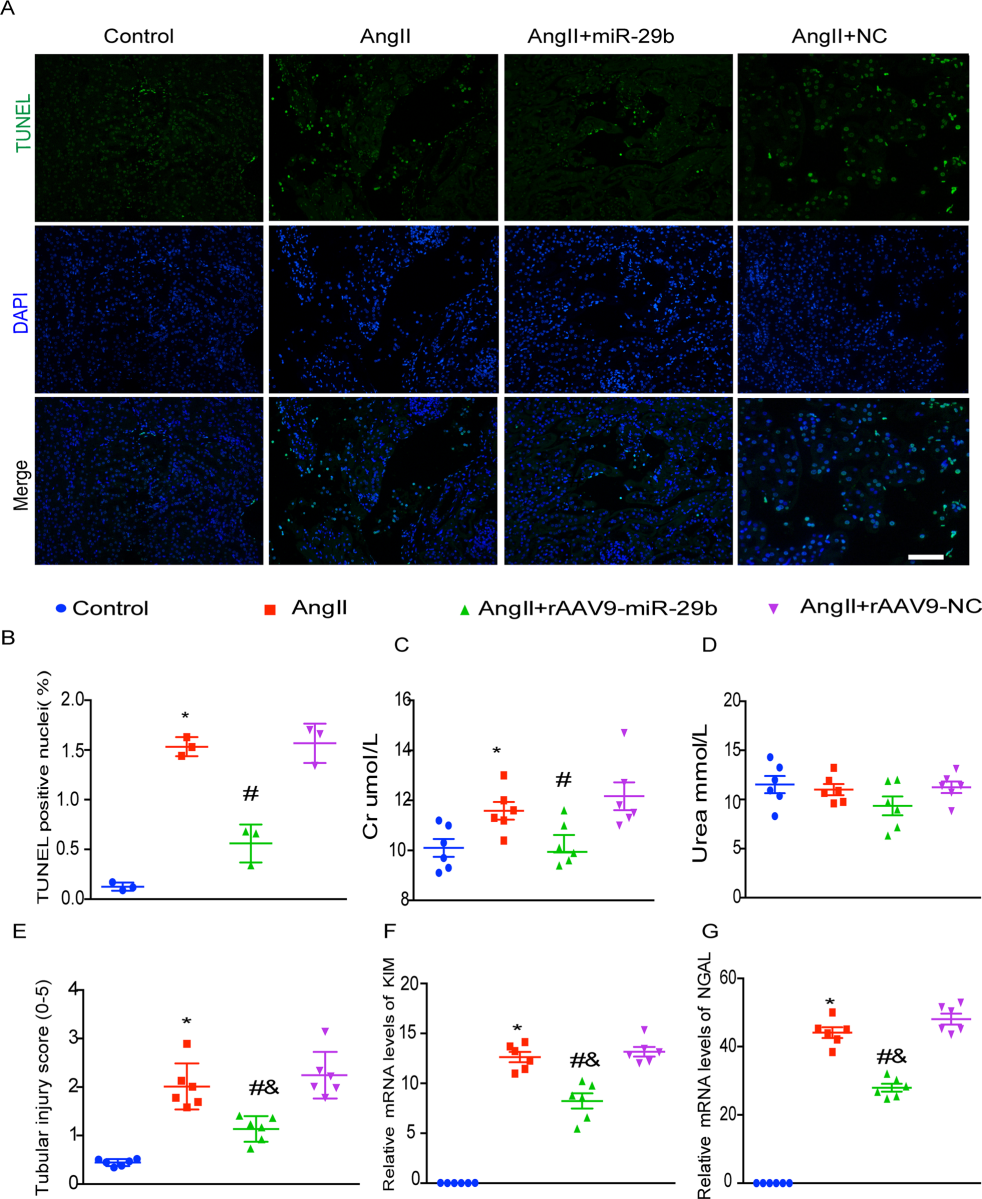
Effects of miR-29b gene transfer on renal injury in vivo. **A** Assessment of apoptosis. Representative epifluorescence images of TUNEL labeling of kidney histological sections at 5 weeks post gene transfer. Scale bar: 50 μm **B**, Quantification of TUNEL-positive nuclei. Values are mean ± SEM measured from 10 000 nuclei (n = 3). **C**, **D**, Renal function was assessed by the level of serum creatinine and urea nitrogen in each group. n = 6. **E**, tubular injury score in each group. **F**, **G** Kidney injury molecule-1 (KIM-1, **E**) and neutrophil gelatinase-associated lipocalin (NGAL, **F**) in renal cortex determined by real-time RT-PCR. mRNA values were normalized by the levels of GAPDH mRNA and then expressed relative to the mean value for the control group (n = 6). Values are mean ± SEM. *P < 0.05 vs. Control; ^#^*P* < 0.05 vs. AngII + rAAV9-Negative control (NC)

rAAV9-miR-29b delivery reduced renal tubular injury in mice. Apoptosis of renal tubular epithelial cells, serum creatinine, urea nitrogen levels and tubular injury score were used to assess tubular injury. Increased miR-29b expression reduced renal tubular injury and attenuated tubular injury parameters in blood (Fig. 5A–E). In summary, miR-29b is closely associated with the fibrotic process, and rAAV9-miR-29 delivery protected tubular epithelial cells.

Genes expression of kidney injury molecule-1 (KIM-1) and Neutrophil gelatinase-associated lipocalin (NGAL), as early predictive markers of renal tubular injury, were used for detecting renal injury. Both mRNA levels of KIM-1 and NGAL in AngII-infused and rAAV9-miR-29b delivery mice were much lower than those in AngII-infused or negative control mice, although they were still higher than those in control mice (Fig. 5F, G).

These results indicate that tubular injury had occurred in Ang II-induced renal fibrosis mice but was much less in rAAV9 mediated miR29b-delivery mice.

## Discussion

The results of our study suggest delivery of rAAV9-miR-29b inhibited renal interstitial fibrosis and injury by suppressing ECM deposition. Therefore, miR-29b plays a pivotal role during the process of renal interstitial fibrosis and may represent a potential therapeutic target for tissue fibrosis. This is the first report to show that delivery of exogenous miR-29b with rAAV9 vector ameliorated renal interstitial fibrosis in vivo. The ability of a single miR to regulate the levels of hundreds of genes (Arash et al. 2012) suggests that modulation of individual miRs can affect multiple signaling pathways. A previous study (Liu et al. 2010) showed that miR-29b may be a master regulator of over 20 collagens and other genes associated with the ECM, which may be relevant to development of renal interstitial fibrosis in rats. Therefore, therapeutically supplementing exogenous miR-29b may alleviate the deleterious effects of ECM deposition associated with renal interstitial fibrosis. However, no significant renal dysfunction occurred in our mouse model.

In the present study, we assessed the effect of persistent rAAV9 vector-mediated miR-29b gene expression in AngII-induced renal interstitial fibrosis in vivo. Our results showed that continuous AngII infusion induced pathological changes characterized by excessive ECM deposition in the renal interstitium. Many ECM-associated genes, such as collagens, and fibrogenic factors, and transforming growth factor (TGF)-β3, are targets of miR-29b. Therefore, pathogenic changes can be reversed significantly by overexpressing miR-29b in the kidneys following AngII infusion. We propose a novel therapeutic strategy for treatment of preexisting fibrosis based on renal delivery of miR-29b. Our results demonstrated that miR-29b supplementation reversed interstitial fibrosis by decreasing ECM deposition. The 24-h urine of mice was collected in metabolic cages, and 24-h urine protein was quantified using the Bradford method. However, no significant difference was found between the scramble group and AngII-infusion group (data not shown). Although histologically, the mice developed renal interstitial fibrosis, this may have been related to the strong resistance of mice to kidney damage. Unlike other models such as obstructive nephropathy, which can cause acute kidney injury, the model of continuous Ang II infusion stimulates RAAS-inappropriate activation. Renal failure may therefore be the result of a long-term evolution process in this type of model.

Our results are consistent with studies that demonstrated the effects of miR-29b in cardiac fibrosis and diabetes (van Rooij et al. 2010). The present study revealed an important aspect of the pivotal role that miR-29b plays in the attenuation of RAAS activation-induced fibrosis, a key pathological feature of kidney remodeling. Some miRs, such as miR-200a, miR-200c, and miR-141, play a key role in pathological remodeling of many organs by targeting various ECM-associated proteins (Xiong et al. 2012). The miR-29 s family was found to play an important role in the fibrotic process in many organs including the kidney, heart, and arteries (Kriegel et al. 2012). Although members of the miR-29 family exhibit diverse biological functions, miR-29 has been implicated to be highly important in the regulation of ECM production, which characterizes the pathological process of renal interstitial fibrosis (Liu et al. 2010).

Consistent with previous findings, our results suggest that miR-29b may serve as a potential therapeutic target for fibrosis because it regulates expression of multiple collagen proteins.

We hypothesized that rescuing miR-29b expression in the mouse kidney reduces renal fibrosis and protects renal function. We used rAAV vector-mediated gene transfer technology to demonstrate that AngII-induced decrease in renal miR-29b expression could be restored by re-expression of miR-29b in mouse kidneys. rAAV vectors provide sustained long-term gene expression with minimal immunological consequences. According to our results, no adverse effects of the rAAV9 vector on renal morphology or function were observed in mice. Therefore, rAAV9 vectors can maintain stable and sustained expression of miRs in vivo without inducing a harmful immune response. rAAV vectors have been applied in some clinical trials (Schievenbusch et al. 2010). Some studies found that, compared with rAAV2 and rAAV8, rAAV9 was the most effective serotype for kidney gene transfer (Schievenbusch et al. 2010; Shen et al. 2018). Compared with other organs, such as the liver and heart, the efficiency of gene transduction in the kidney is lower. Systemic administration of rAAV vector by intravenous injection provides efficient transduction in liver and heart, but provides poor transduction in the kidney. We used in situ injection of rAAV9 vector carrying GFP to determine the magnitude and distribution of gene expression in the kidney. Our results suggested that this method of renal gene delivery is safe and effective. Injection in the kidney did not impair renal function in saline-infused mice.

In our study, we aim to determine whether rAAV9 mediated miR-29b supplementation could improve renal interstitial fibrosis induced by RAAS activation. We didn't measure the blood pressure of mice, which might be the limitation of our study. We focus on the mechanism of ECM position in the process of fibrosis rather than hypertension. Further study need to conduct to investing the regulation of miR-29b in blood pressure.

## Conclusion

Administration of rAAV9 vectors via in situ renal injection achieved safe and effective exogenous gene expression in the kidney. rAAV-mediated supplementation of miR-29 ameliorated interstitial fibrosis and attenuated progressive renal injury through reversal of maladaptive organ remodeling. Restoration of miR-29b levels may serve as a novel therapeutic strategy for reversing renal fibrosis.

## Supplementary Information

**Additional file 1.** A detailed description about the materials and methods.

## Data Availability

Some or all data, models, or used during the study are available and could be cited from the corresponding author by request. (Yu Wang-Qi).

## Abbreviations

ECM: Extracellular matrix
EMT: Epithelial-mesenchymal transition
RAAS: Renin–angiotensin–aldosterone system
miRs: MicroRNAs
MR: Mineralocorticoid receptor
rAAV9: Recombinant adeno-associated virus serotype 9
eGFP: Enhanced green fluorescent protein
CMV: Real time polymerase chain reaction
SEM: Standard error
αSMA: α-Smooth muscle actin
KIM-1: Kidney injury molecule-1
NGAL: Neutrophil gelatinase-associated lipocalin
TGF: Transforming growth factor
