# Supplementary material for: rAAV9‐mediated supplementation of miR-29b improve angiotensin-II induced renal fibrosis in mice
Source: Mol Med. 2021 Aug 18;27:89. doi: 10.1186/s10020-021-00349-5 (PMC8375072; doi:10.1186/s10020-021-00349-5)
Supplement: Supplementary file 1 — Additional file 1. A detailed description about the materials and methods. [file 10020_2021_349_MOESM1_ESM.doc]

**rAAV9‐mediated supplementation of miR-29b improve Angiotensin-II induced Renal Fibrosis in mice**

Zhang Ju-Hong^2^, Jing Li^2^, Ye Yang^2^, Yu Wang-Qi ^1*^

1 The Affiliated Hospital of Hangzhou Normal University, Hangzhou, 310015, Zhejiang, P.R. China.

2 Department of Cardiology, Sir Run Run Shaw Hospital, Zhejiang University, Hangzhou, 310016, Zhejiang, P.R. China.

*Corresponding Author: Wang-Qi Yu

1. E-mail: yuwq13588@163.com

Tel./fax: +86 0571 86006242.

Address: No.1Wenzhou Road, Gong Shu District, Hangzhou, 310016, Zhejiang Province, P.R. China.

Type of manuscript: Original article.

Total number of pages: 14

Number of photographs: 5

Word counts: 3673

**Additional files**

**Methods**

1.1 *Preparation of protein and western blot*

To isolate protein, kidney tissues were immersed in RIPA lysis buffer with [protease and phosphatase inhibitor Cocktail (abcam, ab201119)](https://www.abcam.cn/protease-and-phosphatase-inhibitor-cocktail-ab201119.html), together with beads. Next, tissues were repeatedly disrupted using a homogenizer (at low speed for 30 sec at 4°C per round), and stored on an ice bath for 1 min per round until tissues were completely disrupted. Following centrifugation at 14,000 × *g* for 15 min at 4°C, clear supernatants were transferred to new tubes for the following procedures. Equal amounts of protein were loaded on and separated by 12% SDS-PAGE. Protein was then transferred to PVDF membranes (Bio-Rad, 162-0255). After blocking, membranes were incubated at 4°C for at least 16 h with primary antibodies including anti-GFP and HRP-labeled anti-GAPDH (1:5000, Cell Signalling Technology). Membranes were then washed three times for 5 min in TBST. Next, membranes were incubated at room temperature for 2 h with horseradish peroxidase-conjugated anti-rabbit IgG secondary antibody (1:2500, Cell Signaling Technology). Relative protein levels were analyzed using Image J software and normalized to GAPDH protein levels in the same sample.

1.2 Mous*e model of AngII-induced renal fibrosis*

Mice in three of the groups were treated with continuous AngII (1.44 mg/kg/day) infusion for 28 days using an Alzet micro-osmotic pump (Model 2004; Durect Co., Cupertino, CA, USA). One group received continuous PBS infusion. After 28 days infusion, blood was collected from the orbital cavity. Kidney samples were harvested and immersed in RNA preservation solution or liquid nitrogen. Serum creatinine levels were measured using an automated biochemical analyzer (Beckman Coulter Au5800, Tokyo, Japan) and Accuras Auto CRE kit (Shino-Test Corporation, Tokyo, Japan). Levels of urea nitrogen were measured using a Urease ultraviolet rate kit (Beckman, USA).

1.3 Histology and Immunohistochemistry

To evaluate renal remodeling such as the degree of tubular injury and fibrosis. Samples were mounted on normal glass slides and stained with both Masson trichrome stain and Sirius red stain for collagen deposition examination. For the collagen volume fraction (CVF) analysis in the tubular basement membrane and interstitial space, the percentage of cortex fibrosis was quantified by using the Image J software. Five fields of vision for each slice (magnification, 200×) were selected and analysis of CVF (CVF=collagen stain/ total area of each vision). Samples for sirius red stain visualized under polarized light. We quantified the pixels of type I and type III collagens images using the histogram of Photoshop CC 2017 software.

Tubular injury was defined as tubular dilation, tubular atrophy, sloughing of tubular epithelial cells, or thickening of the tubular basement membrane (*Score 0: no tubular injury; score 1, 10% of tubules injured; score 2, 10%–25% of tubules injured; score3, 26%–50% of tubules injured; score 4, 51%–75% of tubules injured; score 5, .75%*), and the mean was used as the fibrosis score(0-5). Five nonoverlapping fields of renal interstitial area were scored with a semi-quantitative ordinal scale.

*1.4 Terminal deoxynucleotidyl transferase dUTP nick end labeling （TUNEL） assay*

Slides were also deparaffinized and followed by rehydrate in gradient ehtanol of 95%,90%,80% and 70%, respectively. Then slides were washed in distilled water. The objective tissue was marked with liquid blocker pen. Slides were incubated 20 minutes at room temperature after adding permeabilizing working solution and further digested with 1μg/ml proteinase K(Servicebio, G1205) for 25 minutes. Next TUNEL reagents(Roche, 11684817910 ),TdT and dUTP, were mixed at ratio of 1:9 and added to objective tissue. Then slides were placed in a flat wet and incubated for 2h. Then they were mounted with DAPI (Servicebio, G1012). Negative controls for this procedure included a slide without TdT enzyme or pretreated with DNAse I before the normal TUNEL procedure. Photographs of slides were captured by inversed fluorescent microscope (Leica, Germany).

*1.5 Quantitative reverse-transcription polymerase chain reaction (qRT-PCR)*

For miR analysis, to determine the enrichment of mature miR-29b, homogenized kidney specimens were isolated using a miRNeasy Mini Kit (Qiagen, 217004) and 1 ug of each purified RNA was reverse transcribed by using the miScript® II RT Kit (Qiagen, 21861). Real-time qRT-PCR was performed using a miScript SYBR Green PCR Kit (Qiagen, 218073) and the miScript Primer Assay (Qiagen, CAS00006566).The levels of miR-29b were normalized by U6. Data are expressed relative to the control group. For mRNA analysis, reverse-transcription of total RNA was performed using a PrimeScript RT reagent kit with gDNA Eraser (Takara, RR047A) and RNAs were quantified using a SYBR Green RT-PCR Kit (Takara, RR420A). Gene expression levels were quantified by the 2^−∆∆Ct^ method. Real time qPCR conditions were set up according to reagent instructions. *miR-29b* level was quantified using the miScript Primer Assay (Qiagen, CAS00006566). GAPDH and U6 small nuclear RNA was used for normalization. All of the mice primers for the qRT-PCR are listed below.

Collagen I forward: 5’-TGCCGTGACCTCAAGATGTG-3’

Collagen I reverse: 5’-CACAAGCGTGCTGTAGGTGA-3’

Collagen III forward: 5’-GCGGAATTCCTGGACCAAAAGGTGATGCTG-3’

Collagen III reverse: 5’-GCGGGATCCGAGGACCACGTTCCCCATTATG-3’

a-SMA forward: 5’-ACTCTCTTCCAGCCATCTTTCA-3’

a-SMA reverse: 5’-ATAGGTGGTTTCGTGGATGC-3’

KIM-1 forward: 5’-ACATATCGTGGAATCACAACGAC-3’

KIM-1 reverse: 5’-ACTGCTCTTCTGATAGGTGACA-3’

NGAL forward:5’-ATGTCACCTCCATCCTGGTC-3’

NGAL reverse:5’-ACAGCTCCTTGGTTCTTCCA-3’

GAPDH forward: 5′-GCAGTGGCAAAGTGGAGATTG-3′

GAPDH reverse: 5′-AGAGATGATGACCCTTTTGGCTCC-3′

U6 forward: 5’-GCGCGTCGTGAAGCGTTC-3’

U6 reverse: 5’-GTGCAGGGTCCGAGGT-3’
